# Yogurt reintroduction and the circulating microbiome in healthy volunteers: protocol for a prospective, longitudinal, species-controlled crossover clinical trial (MAMI)

**DOI:** 10.1016/j.conctc.2025.101579

**Published:** 2025-11-24

**Authors:** Junhong Su, Bettina E. Hansen, Zifang Wang, Abylaikhan Sharmenov, Xueshan Xia, Michelle Broekhuizen, Zhongren Ma, Maikel P. Peppelenbosch

**Affiliations:** a Department of Gastroenterology and Hepatology, Erasmus MC—University Medical Center Rotterdam, PO Box 2040, NL-3000 CA, Rotterdam, the Netherlands; b Toronto Centre for Liver Disease, University Health Network, & Institute of Health Policy, Management and Evaluation, University of Toronto, Toronto, Canada; c Department of Epidemiology, Erasmus MC—University Medical Center Rotterdam, the Netherlands; d Biomedical Research Center, Northwest Minzu University, Lanzhou, China; e Yunnan Province Key Laboratory of Public Health and Biosafety, School of Public Health, Kunming Medical University, Kunming, China; f Department of Neonatology, Department of Pediatric and Neonatal Intensive Care, Erasmus MC —University Medical Center, Rotterdam, the Netherlands

**Keywords:** Milk consumption, The blood microbiome, The gut microbiome, Healthy volunteers, Milk-associated intestinal bacteria

## Abstract

**Background:**

Although the gut microbiome plays a crucial role for maintaining overall host homeostasis and metabolism, it is significantly influenced by dietary changes, leading to substantial temporal variations in microbial composition within and between individuals. Despite this, incidental fecal sampling remains the standard method for microbiome assessment. Recently, the blood microbiome, defined by microbial DNA (cmDNA) circulating in the bloodstream, has emerged as a potentially more stable and integrated alternative. Preliminary data suggest that blood microbiome analysis may offer more consistent insights than fecal-based approaches, although the methodological validity of the approach has been questioned.

**Method/design:**

This study aims to establish or rule-out cmDNA as a representative of the gut microbiome. In a prospective, single-arm crossover trial, effects of dairy product withdrawal and reintroduction of a yoghurt with a known consortium of bacteria will be assessed in healthy volunteers aged 18–65. Participants will first abstain from all dairy products, a phase expected to reduce yogurt-associated cmDNA in the bloodstream. Yogurt will then be reintroduced, during which reappearance of cmDNA of specific bacteria (especially LGG, LA-5 and BB-12) is anticipated. Shotgun metagenomic sequencing will be used to track cmDNA dynamics over time. This longitudinal sampling approach will provide experimental evidence supporting the existence and responsiveness of the circulating microbiome, while also revalidating the bioinformatic pipeline used for its analysis.

**Conclusion:**

This pilot study will test whether blood-derived microbial DNA can serve as a valid surrogate for gut microbiome composition. If successful, this approach may provide a more stable and integrative alternative to fecal sampling and support future biomarker development and mechanistic research.

**Clinical trial registration:**

NCT06944002.

## 1 Introduction

Ecological DNA (eDNA) is a concept that enables comprehensive descriptions of all organisms present within an ecosystem. The gut microbiomes can be viewed as a specific application of this concept, representing the diverse community of microorganisms inhabiting the gastrointestinal tract [1,2]. In principle, a full description of the gut microbiome should theoretically include all microbial domains, bacteria, viruses, archaea, fungi, as well as other organisms such as worms and phages. However, accurately profiling the gut microbiome remains challenging due to inherent temporal variability and technical limitations. For example, the widely used 16S rRNA gene sequencing method primarily targets bacteria and overlooks other important taxa [3]. Despite these challenges, detailed and accurate characterization is critical, given the gut microbiome's pivotal role in maintaining host health and homeostasis [4,5].

To address the challenges of sampling the intestinal microbiome, most researchers currently rely on fecal analysis. While convenient, this approach has notable limitations. Our team has pioneered advanced technical methodologies, including a double-balloon endoscopic study in healthy volunteers, to comprehensively characterize the gut microbiome in situ [6]. However, fecal samples often fail to accurately reflect the mucosa-associated (adherent) microbiota, which resides in close proximity to epithelial cells and may exert more direct effects on host physiology than luminal bacteria [7,8]. Moreover, fecal microbiomes exhibit considerable heterogeneity, with microbial composition varying across different portions of a single sample. Additional technical barriers include the inefficiency of standard DNA extraction protocols in recovering eukaryotic organisms. Compounding these issues is the strong influence of diet on fecal microbial profiles, distinct microbial communities can emerge following different meals, such as breakfast versus dinner [9]. Collectively, these limitations underscore the need for alternative, more stable and representative sampling strategies capable of generating microbiome data predictive of host health.

An emerging strategy for microbiome analysis involves profiling the blood microbiome, a novel approach that captures microbial signals from multiple body compartments, particularly the gut [10]. The blood microbiome comprises circulating microbial DNA (cmDNA), which consists of fragmented genetic material from microorganisms rather than intact, viable cells. The precise mechanisms through which microbial DNA enters the bloodstream remain unclear [11], though the gastrointestinal tract is considered a major source, especially in the context of impaired gut barrier integrity, a feature commonly observed in chronic diseases. Blood-derived microbiome signatures have demonstrated potential as non-invasive biomarkers for a range of conditions, including diabetes, cardiovascular disease, and cancer, with preliminary evidence suggesting they may even distinguish among different cancer types [10,12]. Despite this promise, the field is subject to ongoing debate, largely due to technical limitations in bioinformatic analysis and the risk of contamination or other artifacts [13,14], underscoring the need for rigorous proof-of-concept studies. These challenges highlight the urgent need for rigorous, well-controlled proof-of-concept studies, a gap that our current research aims to address.

Building on previous findings, we observed that *Lactococcus lactis*, a dairy-dependent intestinal bacterium, is undetectable in human stool within four days of dairy withdrawal, but reappears rapidly upon dairy reintroduction [15,16]. Although the precise half-life of bacterial DNA in human blood is unknown, studies on cancer- and fetal-derived cell-free DNA suggest it is typically less than 24 h [17]. Based on this, a 10-day period of dairy exclusion should markedly reduce cmDNA from milk-associated microbes, with subsequent reemergence following dairy intake.

In this study, we describe the rationale, bacterial-species controlled, and analytical framework for a single-arm crossover trial evaluating the effects of yogurt consumption on the blood microbiome in healthy volunteers. The primary aims are: (1) to assess the presence and abundance of yogurt-dependent intestinal bacterial (YDB) DNA in both blood and fecal samples, and (2) to characterize broader microbiome DNA signatures, including bacteria, viruses, and archaea, in these biological compartments. We hypothesize that yogurt consumption, known to beneficially influence the fecal microbiome and immune function, will induce detectable alterations in cmDNA profiles, reflecting a convergent microbiome signature between the gut and bloodstream. Successfully capturing this phenomenon in a controlled dietary intervention would provide critical proof-of-concept for the presence and dynamic nature of the human blood microbiome.

## 2 Methods

### 2.1 Study design

We will perform a within-subjects crossover trial with a single arm to study the effect of yogurt on the blood and fecal YDB levels in healthy volunteers. Participants will first undergo a 10-day period of dairy product withdrawal. Given that the half-life of bacterial DNA in blood is likely less than 24 h, a 10-day withdrawal period should be sufficient to substantially reduce circulating DNA fragments originating from *L. lactis*. Following the reintroduction of dairy (via yogurt consumption that is host to LGG, LA-5 and BB-12 cultures), we expect these specific bacteria and other YDB DNA to reappear in the bloodstream. While the precise kinetics of this reappearance are not yet known, our study is designed to detect these temporal changes and establish a foundation for understanding the dynamic relationship between dietary intake, the gut microbiome, and the blood microbiome.

This study is approved by the ethics committee of Erasmus Medical University Center Rotterdam in May 2025 (NL88008.078.24) and is registered at www.clinicaltrials.gov (NCT06944002), hence prior to participant's enrollment. This study will be conducted in accordance with the principles of the Declaration of Helsinki (75th WMA General Assembly, Helsinki, Finland, October 2024) and the Medical Research Involving Human Subjects Act (WMO). Interested volunteers will be provided with detailed information about the study and will give written informed consent prior to participation.

**Fig. 1:**
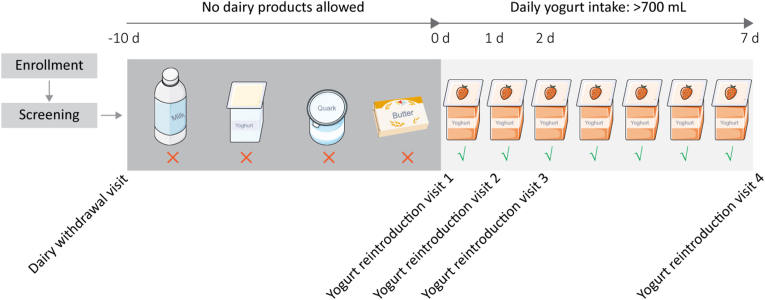
Study design. In this within-person crossover trial, the volunteers will be studied multiple times: a baseline sample of blood and stool will be collected before the withdrawal of any dairy product. Ten days later, the same samples will be collected again to verify if milk and/or yogurt-associated bacteria disappear from the stool and the blood DNA. Subsequently, volunteers will be instructed to drink minimal 700 mL yogurt during breakfast for 7 consecutive days. Three time points will be chosen to capture the dynamics of yogurt-associated DNA gut-to-blood translocation: 24 h, 48 h, and one week after the reintroduction of dairy products. Participants will be invited to visit the hospital for a total of five times. The dairy products used in this study will be provided by the investigational team.

Participants will be studied at multiple time points using a within-subject crossover design (Fig. 1). At baseline, prior to any dietary intervention, blood and stool samples will be collected. Following this, participants will abstain from all dairy products for 10 days, after which a second set of blood and stool samples will be collected to confirm the expected disappearance of YDB DNA from both fecal and circulating microbial DNA pools.

Subsequently, participants will reintroduce dairy into their diet by consuming 700 mL of yogurt daily for seven consecutive days, an amount consistent with recommendations from the United States Dietary Guidelines [18]. Given the current lack of data on the kinetics of bacterial DNA translocation from the gut to the bloodstream, we will collect post-intervention blood and stool samples at three distinct time points: 24 h, 48 h, and one week following the reintroduction of dairy. During this reintroduction phase, participants will also be permitted to consume other dairy products in addition to yogurt.

### 2.2 Participant recruitment

**Fig. 2:**
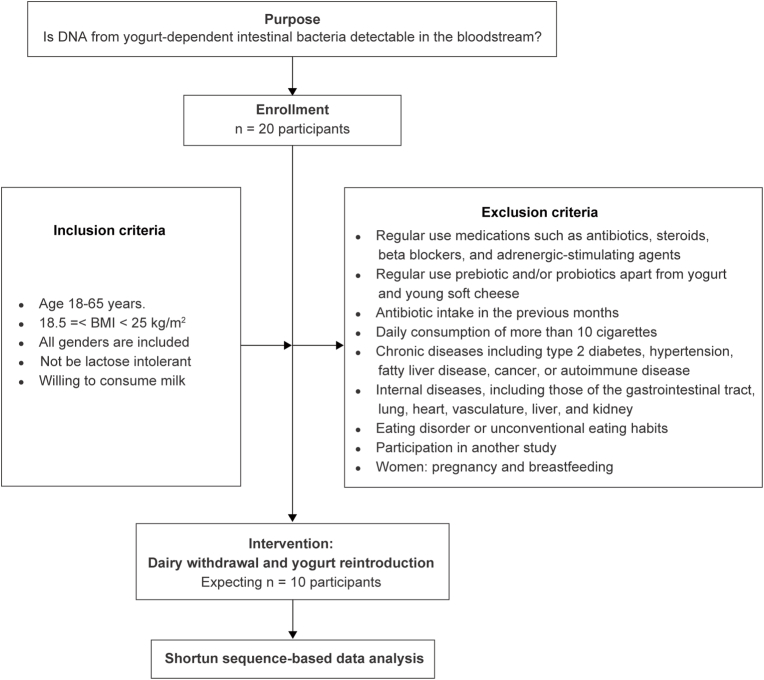
Study schema. This study requires to include data from at least 10 participants.

Healthy volunteers who are interested in participating in the study will be invited. To do this, flyers with contact information (for the secretary) will be posted within the university. All participants expressing interest will receive a study description document that they can read and evaluate at home. Participants will be given one week to make their decision. If they have additional questions, they may consult the leading investigator. One week later, participants will be approached again. After providing sufficient information and answering any questions, participants who agree to participate will be requested by the authorized person to sign the informed consent form and to send it to the coordinating investigator or bring the form to the hospital to sign in the presence of the coordinating investigator. The process of the study is shown in Fig. 2.

### 2.3 Inclusion criteria

1) Age 18–65 years
2) 18.5 =< BMI <25 kg/m2
3) All genders included
4) Not be lactose intolerant
5) Willing to consume milk

### **2.4** **Exclusion criteria** (self-report)

1) Regular use medications such as antibiotics, steroids, beta blockers, and adrenergic-stimulating agents
2) Regular use prebiotic and/or probiotics apart from yogurt and young soft cheese
3) Antibiotic intake in the previous months
4) Daily consumption of more than 10 cigarettes, a minimum quantity known to significantly affect the gut microbiome [19].
5) Chronic diseases including type 2 diabetes, hypertension, fatty liver disease, cancer, or autoimmune disease
6) Internal diseases, including those of the gastrointestinal tract, lung, heart, vasculature, liver, and kidney
7) Eating disorder or unconventional eating habits
8) Participation in another study
9) Women: pregnancy and breastfeeding

### 2.5 Objective

#### 2.5.1 Primary objective

The primary objective is to examine yogurt-dependent bacterial (YDB) DNA counts in fecal and blood samples before and after dairy consumption. YDB, such as *Lactococcus lactis*, are transient residents of the human gut, only present following dairy intake, and therefore represent modifiable components of the intestinal microbiome. Detection of YDB DNA fragments in the bloodstream after dairy reintroduction will provide definitive evidence on the extent to which the blood microbiome reflects relevant changes in the intestinal microbiome. In this specific study, the use of a commercial yoghurt (but verified by independent agar growth testing) containing a defined consortium of bacteria adds signature species whose detection provides convincing signals in this respect.

#### 2.5.2 Secondary objective(s)

To explore changes in the broader microbiome DNA landscape, including bacterial, viral, and archaeal DNA, in both fecal and blood samples before and after dairy consumption. This analysis aims to identify additional microbial signatures that may respond to dietary intervention and assess whether non-YDB microbial DNA in the blood mirrors corresponding shifts in the intestinal microbiome and yogurt consumption-induced effects on immune function.

#### 2.5.3 Long-term objective

The ultimate objective is to develop technology capable of reliably characterizing the dynamics of microbiomes residing in and on the human body. This study will evaluate the extent to which this goal is achievable. It is anticipated that non-dairy-dependent microorganisms will exhibit relatively stable levels over time, whereas dairy-dependent organisms such as *Lactococcus* and *Bifidobacterium* species are expected to show measurable changes. Detecting will be critical for assessing the utility of blood microbiome technology in generating comprehensive, integrative descriptions of human-associated microbiomes.

### 2.6 Power analysis

This is a pilot study. This research has never been conducted before, making it difficult to determine a definite sample size. Based on published studies showing significant change in the relative abundance of gut microbiome at the phylum level upon consumption of fermented milk in 6 volunteers (Firmicutes and Bacteroidetes; p < 0.05 when comparing milk consumption vs. baseline) [20], and that the relative abundance of Bifidobacterium species increased following the consumption of human milk oligosaccharides by 7 volunteers over 7 days (p < 0.05 when comparing four type of milk concentrations with baseline) [21], we have chosen to include 10 volunteers for this study. If this is not sufficient to convincingly demonstrate trends of *Lactococcus lactis* DNA during 7 days of milk consumption intervention, then blood microbiome studies may not be powerful enough to show meaningful changes in intestinal microbiome composition.

### 2.7 Study procedures (Table 1)

#### 2.7.1 Screening

Participants will undergo a medical history assessment and physical examination, including measurements of height, weight, blood pressure, and body mass index (BMI). Medical history will cover past and current diseases, medication use, and smoking status.

#### 2.7.2 Baseline visit for milk withdrawal

Two weeks after the screening, participants will attend a morning baseline visit at the hospital. During this visit, medical history, body composition (weight, waist circumference, and BMI), and blood pressure will be assessed. Blood samples will be collected for blood microbiome analysis, and participants will be asked to provide a fecal sample (Table 2).From this day onward, participants will eliminate all dairy products from their diet for 10 consecutive days. Compliance with diary product withdrawal will be confirmed by direct interrogation at the sampling times, a questionnaire and by detecting YDB in the fecal samples using shotgun sequencing. Participants apparently not dairy-product withdrawn will be excluded from the final analysis and replaced by newly-recruited subjects.

**Table 1:** Overview of study procedures.

	Study period (Days)					
	Enrollment	Milk withdrawal	Intervention			
Time	T = −15∗ day	T = −10 day	D_0_	D_1_	D_2_	D_7_
Suitability screening	X					
Informed consent	X					
Intervention: Milk withdraw → Yogurt reintroduction						
Measurements						
Anthropometric variables	X	X	X	X	X	X
Blood microbiome assessment		X	X	X	X	X
Fecal microbiome assessment		X	X	X	X	X
BMI		X	X	X	X	X
Blood pressure		X	X	X	X	X
Routine laboratory measurements		X	X	X	X	X

Note: D, Days; BMI, body mass index; ∗ will be differed depending on participant's availability.

**Table 2:** Primary, secondary and exploratory outcomes.

Outcomes	Brief description	Time frame
Primary		
Blood MDB	Shotgun sequencing of whole blood DNA	−10, 0, 2, 3 and 7 days
Secondary		
Other blood microbes	Shotgun sequencing of whole blood DNA	−10, 0, 2, 3 and 7 days
The gut microbiome	Shotgun sequencing of fecal DNA	−10, 0, 2, 3 and 7 days
Exploratory		
BMI	Weight in kilograms and height in meters will be combined to report BMI in kg/m^2.	−10, 0, 2, 3 and 7 days
Blood pressure	Systolic and diastolic blood pressure will be assessed with a blood pressure monitor.	−10, 0, 2, 3 and 7 days

Note: MDB, milk-dependent intestinal bacteria.

#### 2.7.3 Pre-intervention visit

On the morning of day 11 (following the 10-day dairy withdrawal period), participants will return to the hospital and provide the same clinical data and biological samples to be collected during the baseline visit. For those who are unable to produce feces on the day of the scheduled visit, sample collection will be permitted either before or after the visit. To avoid confounding baseline measurements, yogurt consumption will only commence after the fecal sample has been collected. After sample collection, participants will begin reintroducing dairy by consuming yogurt for seven consecutive days. To reduce uncertainty regarding bacterial exposure, the number of viable bacteria will be quantified using MRS agar culturing, providing a trackable estimate of bacteria intake. These approaches will ensure microbial consistency across individual participants and minimize batch-related variability. As this is a pilot trial involving no more than 10 participants, it is not powered to assess dosage-dependent effects.

#### 2.7.4 Intervention visit

Participants will attend three additional morning visits during the intervention phase: Day 12 (24 h after reintroducing yogurt); Day 13 (48 h after reintroduction) and Day 18 (after 7 days of yogurt consumption). At each time point, participants will provide the same clinical and biological data as collected during the baseline visit. No follow-up visits are scheduled beyond this point.

### 2.8 Milk dependent microbiome analysis pipeline

#### 2.8.1 DNA extraction and shotgun sequencing

1 Pipeline and experimental controls

**Fig. 3:**
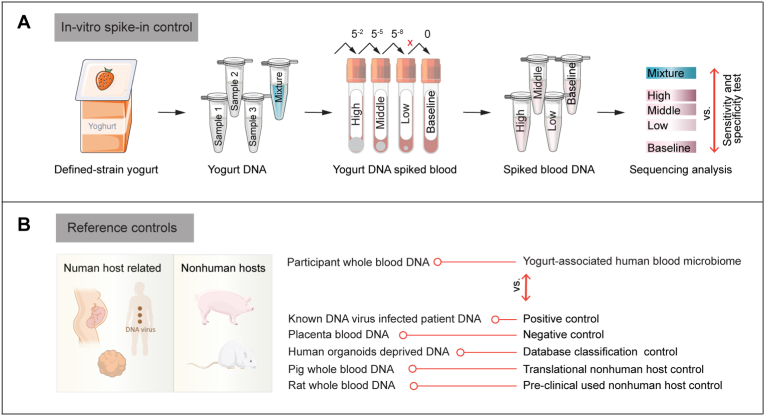
Pipeline designed to validate (circulating microbial DNA) cmDNA as a biomarker of the gut microbiota. A. In-vitro yogurt DNA spike-in control. To assess whether intestinal bacterial DNA enters the circulation and is detectable in the blood, DNA extracted from three commercial yogurt samples of the same batch will be pooled based on concentration, serially diluted, and spiked into participant blood samples (e.g., from the pre-intervention visit) prior to DNA extraction. These spiked samples, together with non-spiked baseline blood DNA sample and pooled yogurt DNA, will undergo shotgun sequencing and taxonomic profiling. This provides both quantitative and qualitative benchmarks for detection sensitivity and specificity. B. Reference controls without the spike in. To explore the existence of a core blood microbiome and distinguish the true microbial signals from background noise, the study includes serval refence controls, which will be processed without added microbial DNA or any artificial spike-in, maintaining its original state to serve as benchmark background signal and classification fidelity: (i) placenta blood as negative control; (ii) blood from patients infected with known viruses as a positive control (blood will be obtained from the Erasmus MC virology department); (iii) human-deprived organoids to assess database classification accuracy and text whether human genomic sequences are misclassified as bacterial in tools like Kraken 2; (iv) whole blood from healthy pigs as a translational non-human host control, based on the hypothesis that pigs may harbor a core cmDNA signature due to dietary and physiological similarities with human. This also reflects the growing relevance of pigs as organ donors in translational research; and (v) rat blood as a preclinical non-human host control, representing a widely used model in experimental and pharmacological studies.

As the central aim of this study is detect yogurt-derived bacterial DNA in blood, we will include an in-vitro control to validate the sensitivity and specificity of our detection pipeline. Blood sample collected at pre-intervention visit, following a 10-day withdrawal from yogurt and all other diary products, will be used for this purpose. Specifically, defined quantities of yogurt DNA will be spiked into participant blood samples prior to DNA extraction. This setup will allow us to confirm that our pipeline can reliably detect yogurt-derived bacterial DNA within a blood matrix (Fig. 3).

To further evaluate the sensitivity and specificity of the entire blood microbiome analysis pipeline, from DNA extraction to data interpretation, a defined microbiome standard (e.g., ZymoBIOMICS Microbial Community DNA Standard) will be included. This benchmark will enable assessment of extraction efficiency, sequencing accuracy and taxonomic profiling cross the pipeline.

Because the blood microbiome may be less than 5 % of the entire blood DNA, interpretation of such low microbial biomass is challenged by potential environment contamination (e.g., lab equipment and kitome) [22,23]. In addition, the definition of a core blood microbiome is further complicated by that the assembled genomes of many bacteria (especially older assemblies) are contaminated by human DNA sequences, leading to false-positive results. The latter issue is considered the biggest problem in the analysis and interpretation of blood microbiomes [14]. The current study, because of its longitudinal character and its reliance on the introduction of defined species with relatively novel assemblies in the database, is designed to provide of proof-of-principle on whether these problems can be overcome. Further, additional power will be added to the current study by introduction of additional controls: we will include a series of reference controls designed to account for all potential sources of contamination and misinterpretation described above (Fig. 3).

2 DNA extraction and shotgun sequencing

To minimize the cross-contamination of kitome from different DNA isolation kits, we will choose the same kits from same company to isolate DNA from all samples types, including feces and blood. The analysis of the cmDNA may follow a slightly modified version of our previously published pipeline [24]. Briefly, fasting blood (5 mL) will be collected by a certified phlebotomist at each time point. Of this, 2.5 ml will be used for DNA extraction and remainder for plasma collection. The samples will be stored at −80 °C until further processing. The blood YDB and other microbial DNA will be assessed by sequencing following the steps below: 1) Blood DNA will be extracted by a commercial available kit or a modified cetyltrimethylammonium bromide (CTAB) method [25]. Briefly, 1000 μl of CTAB lysis buffer at 65 °C with lysozyme will be added to 250 μl of the whole blood and gently inverted. After centrifugation at 12 000 rpm for 10 min, the supernatant will be transferred into a 2.0 ml tube containing phenol/chloroform/isoamyl alcohol (25:24:1). Following another centrifugation, the supernatant will be moved into a new tube, mixed with 24:1 isoamyl alcohol and centrifugated again. The supernatant will be mixed with isopropanol, incubated at −20 °C, and discarded. The precipitate will be washed with 1000 μl of 75 % ethanol twice, dried and dissolved in dd H_2_O. Finally, 1 μl of RNase A will be added, and the mixture will be incubated at 37 °C for 15 min; 2) DNA degradation will be monitored on 1 % agarose gels, and concentration will be measured using Qubit® dsDNA Assay Kit in Qubit® 2.0 Flurometer (Life Technologies, CA, USA). Samples with an OD value between 1.8 and 2.0 and contents above 1ug will be used for library construction; 3) Sequencing libraries will be created using NEBNext® Ultra DNA Library Prep Kit for Illumina (NEB, USA). First, index codes will be added to sequences. Then, DNA sample will be fragmented into approximately 350 bp by sonication, then end-polished, A-tailed, and ligated with adaptors for Illumina sequencing via PCR. Lastly, PCR products with universal primers will be purified (AMPure XP system), with libraries analyzed for size distribution by Agilent2100 Bioanalyzer and quantified using real-time PCR; and 4) The clustering of index-coded samples will be performed on a cBot Cluster system. After cluster generation, DNA library will be sequenced on an Illumina PE150 platform, generating paired-end reads.

#### 2.8.2 Microbiome characterization

1 Blood Lactococcus and other microbial DNA raw data processing

Raw Illumina sequencing reads will be processed using Readfq to remove poor quality bases (threshold ≤38 bp), reads with excessive N bases, and significant adaptor overlaps. Host-derived reads will be filtered using Bowtie2.2.4 software with parameters: -end-to-end, --sensitive, -I 200, -X 400 [26,27].

2 Metagenome assembly

Clean reads will be assembled into Scaffolds using MEGAHIT with -presets meta-large option. Scaffolds will be converted into scaftigs by removing N-containing regions [28,29]. Reads will be mapped back to scaffolds using Bowtie, and fragments <500 bp will be excluded from further analysis [30,31].

3 Gene prediction and abundance calculation

Scaftigs ≥500 bp will be analyzed for open reading frames (ORFs) using MetaGeneMark, filtering out those <100 nt [32,33]. CD-HIT will be used to cluster and remove redundant genes, generating a non-redundant gene catalogue [34,35]. Clean reads will be mapped to this catalogue using Bowtie2, and genes with ≤2 mapped reads will be excluded. Gene abundances will be calculated as described previously [31].

4 Taxonomy prediction

Unigenes will be taxonomically annotated using the Kraken2 database [36], with further validation against the NCBI NR database [37]. The LCA algorithm in MEGAN [38] will be used for accurate species-level assignment. Taxonomic profiles will include relative abundances and gene counts for all taxa, with specific attention to YDB.

#### 2.8.3 Microbiome taxonomic analysis

Krona tool [39] will be used to visualize taxonomic abundance and generate clustered heatmaps. Principal component analysis (PCA) and non-metric multidimensional scaling (NMDS), using the R *vegan* package, will assess variation in microbiome composition. Analysis of similarities (ANOSIM) will test for significant differences between time points. Group-wise taxonomic differences will be identified using LEfSe, with permutation tests and multiple testing correction via the Benjamini–Hochberg false discovery rate. A p-value <0.05 will be considered statistically significant. YDB will be analyzed separately from the pooled shotgun metagenomic dataset due to its specific study relevance.

#### 2.8.4 Assessment of the gut Lactococcus DNA and other microbial DNA

Fecal samples will be collected from all participant at each time point. Faecal DNA will be isolated using the same method as previously described for blood DNA isolation. YDB DNA from feces will be processed and analyzed using the same pipeline as for blood-derived DNA.

### 2.9 Statistical analysis

The primary outcome is the change in relative abundance of blood YDB DNA across five time points. This will be analyzed using repeated measures ANOVA to detect statistically significant within-subject changes over time. The secondary outcomes include changes in all other non-human microbial DNA, which will also be analyzed using repeated measures ANOVA. All statistical tests will be two-tailed, with significance set at p < 0.05.

## 3 Discussion

The existence of a common core blood microbiome in humans remains a subject of debate [10,13]. Most previous studies are cross-sectional and often lack rigorous controls for environmental contamination and database misclassification, which poses a significant challenge and hinders progress in the field. Another major limitation is the absence of longitudinal within-subject controls, which are essential for identifying and correcting potential false positives or negatives caused by individual variability and limitations in bioinformatics pipelines [14]. Furthermore, while the gut microbiome is frequently proposed as the primary source of blood microbial DNA, this hypothesis has not yet been definitively proven.

To overcome these challenges, a longitudinal design using a safe, well-characterized intervention that reliably alters the gut microbiome offers a promising solution. Yogurt, generally regarded as safe and nutritional beneficial, increase levels of beneficial bacteria such as *Lactococcus* and *Lactobacillus*, which are closely linked to immune function [40]. Given the established translocation of gut microbiome-derived metabolites, it is plausible that microbial DNA from yogurt-sensitive gut bacteria could also enter circulation and contribute to the composition of the core blood microbiome. In addition to its microbiome-modulating effects, yogurt provides essential vitamins and minerals such as riboflavin, zinc, selenium, and magnesium that support immune health as well. These combined advantages make yogurt a suitable, targeted and practical modulator for studying the presence and behavior of YDB DNA in circulation.

If cmDNA in blood correlates with the fecal microbiome, the use of defined probiotic strains in yogurt as spike-in controls may offer evidence supporting cm DNA as a valid representation of the colonic microbial composition of the participants involved. Importantly, while these introduced species primarily serve as a proof-of-principle to demonstrate detectability in blood, comprehensive microbiome profiling of fecal material will allow direct correlation analyses with respect to the amount of these bacteria in the volunteer intestine. Thus substantial insight into the relationship between gut and blood microbial signals is expected to be gained in this respect. Should proof-of-principle be established, a future dose response study will allow making more precise quantification and validation of these correlations.

By analyzing YDB DNA in both blood and fecal samples before and after yogurt reintroduction, this study aims to assess the responsiveness of the blood microbiome to dietary changes. If successful, the results could validate our blood microbiome technology and establish a foundation for non-invasive microbiome monitoring. These findings may offer important insights into host–microbiome interactions and contribute to the development of novel diagnostic tools for health monitoring and disease prevention.

## CRediT authorship contribution statement

**Junhong Su:** Writing – review & editing, Writing – original draft, Visualization, Software, Methodology, Conceptualization, Investigation. **Bettina E. Hansen:** Writing – review & editing, Software, Methodology, Investigation. **Zifang Wang:** Writing – review & editing, Methodology. **Abylaikhan Sharmenov:** Writing – review & editing, Resources. **Xueshan Xia:** Writing – review & editing. **Michelle Broekhuizen:** Resources, Writing – review & editing. **Zhongren Ma:** Writing – review & editing, Project administration, Conceptualization. **Maikel P. Peppelenbosch:** Writing – review & editing, Writing – original draft, Supervision, Project administration, Methodology, Funding acquisition, Conceptualization, Investigation.

## Funding

This project is funded in part through a grant (KICH2.V4P.22.015) of the Dutch Organization for Scientific Research and the Dutch Cancer Foundation.

## Declaration of competing interest

The authors declare the following financial interests/personal relationships which may be considered as potential competing interests:Maikel Peppelenbosch reports financial support was provided by The Foundation for Liver and Gastrointestinal Research. Maikel Peppelenbosch reports financial support was provided by Dutch Research Council. Maikel Peppelenbosch reports financial support was provided by Hualing Dairy Co. LTD. Maikel Peppelenbosch reports financial support was provided by Netherlands Organisation for Health Research and Development. Maikel Peppelenbosch reports a relationship with Pfizer Inc that includes: speaking and lecture fees. Maikel Peppelenbosch reports a relationship with Netherlands Organisation for Health Research and Development that includes: consulting or advisory. Maikel Peppelenbosch reports a relationship with Narodowe Centrum Nauki that includes: consulting or advisory. Maikel Peppelenbosch reports a relationship with Czech Science Foundation that includes: consulting or advisory. Maikel Peppelenbosch reports a relationship with European Commission (Brussel) that includes: consulting or advisory. Maikel Peppelenbosch reports a relationship with European Science Foundation that includes: consulting or advisory. Maikel Peppelenbosch reports a relationship with VVD that includes: consulting or advisory. Maikel Peppelenbosch is director of the Batavian Society of Experimental Philosophy and has an appointment as a lecturer at the Technical University of Delft. He is also member of the editorial board of Journal of Crohn's and Colitis, Cells and Biology If there are other authors, they declare that they have no known competing financial interests or personal relationships that could have appeared to influence the work reported in this paper.

## Data Availability

No data was used for the research described in the article.
